# Editorial: Inflammation in muscular dystrophies: mediators, mechanisms, and therapeutics

**DOI:** 10.3389/fimmu.2024.1470266

**Published:** 2024-08-22

**Authors:** Chiara Villa, Andrea Farini, Yvan Torrente

**Affiliations:** ^1^ Stem Cell Laboratory, Dino Ferrari Center, Department of Pathophysiology and Transplantation, Università degli Studi di Milano, Milan, Italy; ^2^ Neurology Unit, Fondazione IRCCS Ca’ Granda Ospedale Maggiore Policlinico, Milan, Italy

**Keywords:** miRNA, DMD, muscular dystrophies, inflammation, GHS (growth hormone secretagogues), NLRP3

The search for effective therapies to treat muscular dystrophies, particularly Duchenne muscular dystrophy (DMD), has been a persistent and formidable challenge. This research is motivated by the urgent need to address the complex pathophysiology of these debilitating diseases, which significantly compromise patients’ quality of life and lifespan.

DMD, in particular, poses a substantial challenge due to its progressive nature and severe impairment of skeletal muscle function. The genetic etiology of the disease, characterized by mutations in the dystrophin gene, initiates a cascade of pathological events, including chronic inflammation.

This Research Topic provides innovative therapeutic strategies targeting inflammation in muscular dystrophies, including the exploration of innate immunity, the therapeutic potential of growth hormone secretagogues, the underlying mechanisms of inflammation-induced muscle atrophy, and the regenerative capabilities of extracellular vesicle-derived miRNAs.

Petrof et al. present a compelling case for the involvement of trained immunity in the pathogenesis of DMD, emphasizing the role of dysregulated inflammation mediated by innate immune cells. Their findings establish a novel framework wherein epigenetic and metabolic alterations induce a hyper-responsive state in innate immune cells, potentially exacerbating tissue damage in DMD.

Complementing this perspective, Boccanegra et al. provide preclinical evidence supporting the therapeutic potential of growth hormone secretagogues (GHS) in DMD. GHSs have demonstrated efficacy in attenuating key drivers of disease progression such as inflammation and fibrosis, and concomitantly exhibited beneficial effects on muscle function and metabolism, suggesting a multifaceted therapeutic potential for improving the quality of life in DMD patients.

In parallel, Liu et al. explore the intricate relationship between inflammation and skeletal muscle atrophy, particularly in the context of sepsis-induced complications. Their investigation of the NLRP3 inflammasome reveals a critical role in driving catabolic processes, implicating it as a potential target for mitigating muscle wasting and associated comorbidities.

Finally, Yedigaryan et al. highlight the therapeutic potential of extracellular vesicle-derived miRNAs in muscle wasting conditions. Through a comprehensive analysis, they identify a hypertrophic miRNA signature capable of enhancing myogenic capacity and attenuating muscle degeneration. This promising approach holds significant implications to stimulate muscle repair and function.

In conclusion, the presented studies underscore the imperative for a multidisciplinary approach to address the challenges posed by DMD. The synergy between basic research, clinical studies, and drug development is essential for identifying novel therapeutic strategies and improving patient quality of life.

